# Long-term training on sand changes lower limb muscle activities during running in runners with over-pronated feet

**DOI:** 10.1186/s12938-021-00955-8

**Published:** 2021-11-27

**Authors:** AmirAli Jafarnezhadgero, Amir Fatollahi, Ali Sheykholeslami, Valdeci Carlos Dionisio, Mohammad Akrami

**Affiliations:** 1 Department of Sports Management and Biomechanics, Faculty of Educational Sciences and Psychology, University of Mohaghegh Ardabili, Ardabil, Iran; 2 Department of Counseling, Faculty of Educational Sciences and Psychology, University of Mohaghegh Ardabili, Ardabil, Iran; 3 Physical Education and Physiotherapy Faculty, Federal University of Uberlândia, Uberlândia, Brazil; 4 Department of Engineering, University of Exeter, Exeter, EX4 4QF UK

**Keywords:** Flat feet, Lower limb mechanics, Unstable walkway, Electromyography, Running

## Abstract

**Background:**

Running on sand could be a promising exercise intervention for the treatment of over-pronated feet. However, there is a lack of knowledge about the effects of running on sand on muscle activities. Therefore, this study aims to evaluate the long-term effects of running on sand on the activities of selected lower limb muscles in individuals with OPF compared with healthy controls.

**Methods:**

Sixty recreational adult male runners with over-pronated feet (foot posture index > 10) were divided into two equal groups (intervention and control). Participants ran barefoot at a pre-defined speed (⁓3.3 m/s) over level stable ground both before and after long-term training on the sand. Muscle activities were recorded using a surface bipolar electromyography system.

**Results:**

For the intervention group, we found a reduced foot posture index (*p* < 0.001; *d* = 2.00) and significant group-by-time interactions for gluteus medius activity during the mid-stance phase (*p* < 0.028; *d* = 0.59). Significantly higher gluteus medius activity (*p* = 0.028, *d* = 0.569) was found during the post-test. We also observed significant group-by-time interactions for medial gastrocnemius activity during the push-off phase (*p* < 0.041; *d* = 0.54). Significantly larger medial gastrocnemius activity (*p* = 0.041; *d* = 0.636) was found during the post-test compared to the pre-test.

**Conclusions:**

Long-term running on sand resulted in reduced pronation, increased medial gastrocnemius activity, and improved frontal plane pelvic stability due to higher gluteus medius activity.

*Trial registration*: IRCT20191211045704N1. Registered 25 February 2020. Retrospectively registered.

## Background

Injuries are a major and ongoing inconvenience of running [1]. Incidence rates of running-related injuries (RRIs) can fluctuate from 3 to 85% [2, 3] and 2.5–33 injuries across 1000 h of activity [4], depending on the population studied. About 87% of RRIs occur in the knee, lower leg, foot, and ankle, with abnormal foot posture being a leading cause of those injuries [5, 6]. The foot is the only part of the body in contact with the ground while running. Therefore, it is responsible for shock absorption and dispersal of ground reaction force across the foot [7, 8]. An atypical medial foot arch can disrupt the shock absorption and attenuation, imposing more stress on the foot or other structures or joints [9]. Hence, abnormality in the foot has the potential to predispose runners to injuries.

Over-pronated foot (OPF) is the most common functional foot abnormality, with a 2‒23% prevalence rate in adults [10–12]. The deficit in muscular strength, lower limb anatomical alignment, and ligament function have been reported as causes of OPF. It was proposed that the dynamic stabilization of the medial longitudinal arch is reliant on the activity of several muscles, including triceps surae, peroneals, tibialis posterior, and tibialis anterior [5, 8]. There is evidence that individuals with OPF demonstrate the increased activity of some leg muscles (tibialis posterior, tibialis anterior, toe flexors, medial gastrocnemius, and gluteus medius) and decreased activation of evertor musculature compared to those with normal feet (5, 13, 14, 15). In addition, a previous study reported higher vastus lateralis and vastus medialis muscle activities in healthy controls compared with individuals with PF during drop landing [16]. Accordingly, previous studies [5, 13, 16] recommended that lower limb muscle activities should be realized during exercise and treatment of individuals with OPF. OPF has been reported as one of the main risk factors predisposing runners to injuries, such as medial tibial stress syndrome, Achilles tendinopathy, and plantar fasciitis [9]. In addition, recently published studies reported that OPF posture is associated with higher odds of RRIs than a normal foot posture [17, 18]. Therefore, modifying OPF may be a possible way of reducing and preventing RRIs.

Different methods have been utilized in the treatment of OPF. Passive supports such as foot orthoses [19, 20], taping [21, 22, 23], and motion control footwear [24, 25] were suggested in the treatment of OPF. However, therapists often prescribe active exercise interventions and confer additional advantages over passive supports because of the improved foot arches brought about by the strengthening of core foot muscles (intrinsic muscles) [26, 27]. Surprisingly, there is a lack of knowledge about the effects of active interventions on muscle activities in individuals with OPF.

Athletes and coaches have considered sand running an excellent way to complement regular training on a firm surface [28]. From a biomechanical perspective, sand running yields greater net knee extensor activity than stable ground [29]. Examining ground reaction forces (GRFs) and muscle activity while sand walking, Jafarnezhadgero et al. observed that sand walking results in lower peak positive free moments (FM) and loading rate compared with stable ground walking [8]. Furthermore, there is evidence that sand running compared with stable ground running impacts kinematics and kinetics in healthy and diseased individuals [29, 30]. Therefore, it can be postulated that sand running changes biomechanical factors, muscle activities and modifies OPF [8, 29].

The originality of this study is due to the duration of the training for individuals with OPF. Although Jafarnezhadgero et al. evaluated GRFs and muscle activity in individuals with OPF compared with healthy control ones while sand walking [8], these authors only evaluated the acute effect (and not long term) of walking on sand versus walking on a stable surface in individuals with OPF [8]. There is currently a lack of evidence investigating the long-term effects of training on sand and the implications for firm ground performance gains in individuals with OPF. Therefore, we aim to evaluate the long-term effects of sand running on activities of selected lower limb muscles in individuals with OPF. It has been demonstrated that during running on sand, electromyography (EMG) in the gastrocnemius, hamstrings (semimembranosus and biceps femoris), vastii (vastus lateralis and vastus medialis), rectus femoris, and tensor fascia latae were greater than the firm surface measures [29]. Another study indicated that more muscles are mobilized during walking on sand surfaces due to reduced surface elastic energy [31]. Concerning the relevant literature [8, 29, 31], we hypothesized different lower limb muscle activities after long-term sand running in recreational adult male runners with OPF.

## Results

No significant between-group differences were found at baseline for all the examined variables (Table 1), but the foot posture index was reduced (*p* < 0.001; *d* = 2.00) after the running on sand training protocol for the IG (pre: 11.2 ± 0.7; post: 9.6 ± 0.9) compared with the CG (pre: 11.0 ± 0.7; post: 11.1 ± 0.7).

**Table 1:** Baseline values of demographic and muscular activity data for all groups

Characteristics	Control	Intervention	p-value
Parameters			
Age (years)	22.2 ± 1.9	22.2 ± 2.5	0.955
Heigh (cm)	177.9 ± 5.7	178.0 ± 6.6	0.869
Mass (kg)	75.40 ± 7.9	75.0 ± 8.2	0.612
Navicular drop (mm)	16.3 ± 1.7	16.2 ± 1.6	0.816
Foot posture index	11.0 ± 0.7	11.2 ± 0.7	0.401
EMG			
Loading phase			
TA	34.67 ± 10.23	34.29 ± 10.91	0.889
Gas-M	15.20 ± 3.66	15.48 ± 4.77	0.795
VL	15.87 ± 12.33	15.55 ± 8.97	0.909
VM	19.09 ± 10.08	19.36 ± 10.28	0.920
RF	20.38 ± 7.34	20.79 ± 10.69	0.865
BF	20.94 ± 11.94	25.61 ± 11.81	0.133
ST	15.96 ± 7.03	15.55 ± 5.97	0.807
Glut-M	31.01 ± 14.34	31.36 ± 11.30	0.915
Mid stance phase			
TA	25.95 ± 11.10	26.26 ± 11.75	0.917
Gas-M	51.09 ± 16.55	51.98 ± 17.47	0.840
VL	46.50 ± 18.53	46.84 ± 18.91	0.945
VM	55.70 ± 26.89	53.56 ± 21.21	0.733
RF	36.51 ± 13.52	37.88 ± 16.51	0.726
BF	23.93 ± 13.63	24.13 ± 11.25	0.951
ST	20.18 ± 9.28	21.50 ± 8.09	0.561
Glut-M	42.06 ± 14.79	42.15 ± 12.38	0.981
Push off			
TA	17.13 ± 9.14	17.17 ± 7.69	0.986
Gas-M	54.85 ± 16.04	54.50 ± 16.95	0.933
VL	25.73 ± 8.59	26.45 ± 9.88	0.764
VM	19.64 ± 5.77	20.07 ± 6.41	0.785
RF	20.35 ± 5.77	20.92 ± 5.37	0.695
BF	19.14 ± 7.93	19.98 ± 7.28	0.671
ST	11.84 ± 5.54	11.72 ± 4.73	0.927
Glut-M	24.59 ± 14.12	24.57 ± 11.74	0.995
Swing phase			
TA	29.31 ± 11.42	28.67 ± 14.65	0.851
Gas-M	11.26 ± 5.05	11.27 ± 4.49	0.996
VL	20.59 ± 9.43	20.58 ± 8.90	0.997
VM	23.50 ± 10.68	23.45 ± 9.53	0.986
RF	19.85 ± 7.28	19.99 ± 5.81	0.935
BF	14.60 ± 8.91	13.27 ± 6.43	0.512
ST	14.39 ± 7.81	14.11 ± 7.57	0.887
Glut-M	23.35 ± 8.73	22.81 ± 8.22	0.807

*EMG* electromyography, *TA* tibialis anterior, *Gas-M* gastrocnemius medialis, *BF* biceps femoris; ST, semitendinosus, *VL* vastus lateralis, *VM* vastus medialis, *RF* rectus femoris, *Glut-M* gluteus medius; P-value from independent samples t-test, *SD* standard deviation

Regarding the EMG activity of the selected lower limb muscles during the loading phase, there was no differential effect of “group by time interactions” (*p* > 0.05; *d* = 0.00‒0.41), nor was there an effect of “time” (*p* > 0.05; *d* = 0.00‒0.40), or an effect of “group” (*p* > 0.05; *d* = 0.00‒0.41) (Table 2).

**Table 2:** Muscle activity pre and post-test for both groups during the loading phase (% maximum voluntary isometric contraction)

Muscles	Control			∆%	Intervention			∆%	Sig. (Effect size)		
	Pre-test	Post-test	95% CI		Pre-test	Post-test	95% CI		Time	Group	Group x Time
TA	34.67 ± 10.23	34.64 ± 8.82	− 2.63,2.69	− 0.08	34.29 ± 10.91	37.10 ± 14.11	− 5.38,− 0.24	8.19	0.129(0.403)	0.706(0.090)	0.121(0.414)
Gas-M	15.20 ± 3.66	15.09 ± 3.65	− 1.32,.1.53	− 0.72	15.48 ± 4.77	15.74 ± 4.25	− 2.48,1.96	1.67	0.907(0.000)	0.579(0.142)	0.780(0.063)
VL	15.87 ± 12.33	15.97 ± 8.90	− 4.54,4.34	0.63	15.55 ± 8.97	16.66 ± 10.65	− 5.08,2.86	7.13	0.680(0.110)	0.934(0.000)	0.731(0.090)
VM	19.09 ± 10.08	18.80 ± 8.58	− 2.13,2.71	− 1.51	19.36 ± 10.28	21.15 ± 9.94	− 6.12,2.55	9.24	0.540(0.168)	0.556(0.155)	0.396(0.220)
RF	20.38 ± 7.34	20.28 ± 6.80	− 2.50,2.70	− 0.49	20.79 ± 10.69	20.44 ± 7.28	− 2.84,3.52	− 1.68	0.828(0.063)	0.880(0.000)	0.905(0.000)
BF	20.94 ± 11.94	20.43 ± 12.69	− 2.27,3.29	− 2.43	25.61 ± 11.81	25.31 ± 12.51	− 3.04,3.62	− 1.17	0.707(0.090)	0.114(0.419)	0.920(0.000)
ST	15.96 ± 7.03	15.49 ± 6.86	− 1.41,2.36	− 2.94	15.55 ± 5.97	16.05 ± 6.48	− 2.67,1.66	3.21	0.981(0.000)	0.962(0.000)	0.490(0.180)
Glut-M	31.01 ± 14.34	30.36 ± 17.06	− 5.69,6.98	− 2.09	31.36 ± 11.30	32.10 ± 10.93	− 5.25,3.75	2.35	0.979(0.000)	0.724(0.090)	0.716(0.090)

*PF* pronated feet, *TA* tibialis anterior, *Gas-M* gastrocnemius medialis, *BF* biceps femoris, *ST* semitendinosus, *VL* vastus lateralis, *VM* vastus medialis, *RF* rectus femoris, *Glut-M* gluteus medius, *CI* confidence interval, *Sig.* Significant level

For EMG activity of the selected lower limb muscles during the mid-stance phase, there was a significant effect of “group-by-time interactions” for Glut-M activity (*p* < 0.028; *d* = 0.59). In the IG, significantly higher Glut-M activity (*p* = 0.028, *d* = 0.569) was found in the post-test compared to the pre-test (Table 3). However, there were no differential effects of “time” (*p* > 0.05; *d* = 0.00‒0.51), nor was there an effect of “group” (*p* > 0.05; *d* = 0.00‒0.37).

**Table 3:** Muscle activity pre and post-test for both groups during the mid-stance phase (% maximum voluntary isometric contraction)

Muscles	Control			∆%	Intervention			∆%	Sig. (Effect size)		
	Pre-test	Post-test	95% CI		Pre-test	Post-test	95% CI		Time	Group	Group x Time
TA	25.95 ± 11.10	26.67 ± 13.06	− 5.05,3.61	2.77	26.26 ± 11.75	26.94 ± 9.24	− 3.57,2.20	2.58	0.584(0.142)	0.913(0.000)	0.988(0.000)
Gas-M	51.09 ± 16.55	50.01 ± 13.46	− 4.29,6.45	− 2.11	51.98 ± 17.47	50.89 ± 13.52	− 3.57,5.76	− 2.09	0.535(0.168)	0.805(0.201)	0.997(0.000)
VL	46.50 ± 18.53	47.42 ± 19.07	− 8.17,6.34	1.97	46.84 ± 18.91	47.14 ± 19.95	− 6.64,6.05	0.64	0.799(0.063)	0.995(0.000)	0.896(0.000)
VM	55.70 ± 26.89	54.51 ± 21.49	− 9.75,12.14	− 2.13	53.56 ± 21.21	59.93 ± 26.16	− 17.96,5.22	11.89	0.510(0.180)	0.736(0.090)	0.336(0.255)
RF	36.51 ± 13.52	36.78 ± 16.77	− 3.83,3.29	0.73	37.88 ± 16.51	40.41 ± 18.23	− 8.90,3.85	6.67	0.438(0.201)	0.516(0.168)	0.530(0.169)
BF	23.93 ± 13.63	23.25 ± 11.02	− 4.37,5.73	− 2.84	24.13 ± 11.25	24.63 ± 10.96	− 4.04,3.05	2.07	0.952(0.000)	0.766(0.090)	0.698(0.110)
ST	20.18 ± 9.28	19.73 ± 9.64	− 3.84,4.76	− 2.22	21.50 ± 8.09	22.15 ± 7.88	− 4.59,3.29	3.02	0.944(0.000)	0.292(0.278)	0.701(0.110)
Glut-M	42.06 ± 14.79	41.42 ± 15.20	− 3.94,5.23	− 1.52	42.15 ± 12.38	50.90 ± 18.33	− 15.94, − 1.54	20.75	0.057(0.510)	0.161(0.375)	0.028(0.590)*

*PF* pronated feet, *TA* tibialis anterior, *Gas-M* gastrocnemius medialis, *BF* biceps femoris, *ST* semitendinosus, *VL* vastus lateralis, *VM* vastus medialis, *RF* rectus femoris, *Glut-M* gluteus medius, *CI* confidence interval, *Sig.* Significant level

* symbol indicate significant difference

For EMG activity of the selected lower limb muscles during the push-off phase, we observed a significant effect of “group-by-time interactions” for Gas-M activity (*p* < 0.041; *d* = 0.54) (Table 4). In the IG, significantly greater Gas-M activity (*p* = 0.041; *d* = 0.636) was found during the post-test compared to the pre-test (Table 4). We also observed that there was no differential effect of “group” (*p* > 0.05; *d* = 0.00‒0.35), but there were significant main effects of “time” for Gas-M activity (*p* < 0.030; *d* = 0.58). Pairwise comparisons revealed significantly greater Gas-M activity (*p* = 0.030; *d* = 0.47) in the post-test compared with the pre-test (Table 4).

**Table 4:** Muscle activity pre and post-test for both groups during the push-off phase (% maximum voluntary isometric contraction)

Muscles	Control			∆%	Intervention			∆%	Sig. (Effect size)		
	Pre-test	Post-test	95% CI		Pre-test	Post-test	95% CI		Time	Group	Group x Time
TA	17.13 ± 9.14	16.36 ± 8.27	− 1.73,3.28	− 3.09	17.17 ± 7.69	17.74 ± 7.39	− 3.34,2.19	3.31	0.915(0.000)	0.708(0.090)	0.465(0.191)
Gas-M	54.85 ± 16.04	55.22 ± 20.45	− 6.85,6.12	0.67	54.50 ± 16.95	66.09 ± 19.47	− 20.49,− 2.69	21.26	0.030(0.582)*	0.182(0.358)	0.041(0.549)*
VL	25.73 ± 8.59	25.39 ± 10.65	− 3.48,4.16	− 1.32	26.45 ± 9.88	27.03 ± 9.71	− 5.45,4.31	2.19	0.939(0.000)	0.559(0.155)	0.764(0.090)
VM	19.64 ± 5.77	19.41 ± 8.07	− 3.02,3.49	− 1.17	20.07 ± 6.41	20.93 ± 8.47	− 4.04,2.32	4.28	0.779(0.063)	0.520(0.168)	0.625(0.127)
RF	20.35 ± 5.77	21.19 ± 8.96	− 4.16,2.48	4.12	20.92 ± 5.37	20.86 ± 5.08	− 2.67,2.79	− 0.28	0.712(0.090)	0.927(0.000)	0.672(0.110)
BF	19.14 ± 7.93	19.37 ± 8.80	− 3.82,3.37	1.20	19.98 ± 7.28	19.03 ± 7.01	− 1.53,3.45	− 4.75	0.733(0.090)	0.855(0.000)	0.583(0.142)
ST	11.84 ± 5.54	11.49 ± 6.11	− 2.46,3.16	− 2.95	11.72 ± 4.73	12.32 ± 6.82	− 3.75,2.54	5.11	0.901(0.000)	0.749(0.090)	0.645(0.127)
Glut-M	24.59 ± 14.12	24.31 ± 11.46	− 2.45,3.01	− 1.13	24.57 ± 11.74	26.85 ± 11.60	− 6.10,1.54	9.27	0.388(0.230)	0.671(0.110)	0.270(0.293)

*PF* pronated feet, *TA* tibialis anterior, *Gas-M* gastrocnemius medialis, *BF* biceps femoris, *ST* semitendinosus, *VL* vastus lateralis, *VM* vastus medialis, *RF* rectus femoris, *Glut-M* gluteus medius, *CI* confidence interval, *Sig.* Significant level

* symbol indicate significant difference

In summary, after the intervention, our results showed increased EMG signals of Glut-M and Gas-M during mid-stance and push-off phases, respectively.

## Discussion

This study aimed to evaluate the long-term effects of sand running on selected lower limb muscle activities in individuals with OPF. The study was the first to evaluate the long-term effects of sand running on active male adults with OPF. Our results highlight sand running as a basis for modifying the foot posture index, Glut-M, and Gas-M activities. The intervention was able to reduce the foot posture index for the IG. We have not measured the EMG activities or strength of intrinsic foot muscles; however, we can assume that the intrinsic foot muscles were strengthened. In a pronated foot, abnormal alignment may stretch and weaken the intrinsic foot muscles by elongating them beyond their neutral physiological resting position. In addition, the alignment changes the length-tension relationship of the muscles, which may preclude the muscle from generating sufficient or optimal force. Various methods have been advocated in treating a pronated foot, including active strengthening exercises [32, 33, 34]. Several active exercises can be used to strengthen the intrinsic foot muscles, reduce foot pronation, and raise the medial longitudinal arch, such as picking up objects, engaging in unilateral balance activities, and performing shin curls, towel toe curls, and the short foot exercise [32, 34].

Short foot exercise is frequently prescribed and performed in sports and rehabilitation to strengthen the intrinsic foot muscles and enhance the longitudinal and transverse arches. A study reported that the short foot exercise (e.g., sand training) was more effective than towel toe curls in activating the abductor hallucis muscles and preventing a lowered medial longitudinal arch [26]. A previous study also observed that the intrinsic strength of the foot increased as the foot posture index was reduced [35]. The improved strength of the intrinsic muscles might also improve the energy transfer across the lower limb [36]. The training program includes six exercises. From our study design, it is unclear exactly which exercise affected EMG and foot posture index changes. Further study is warranted to better established this issue.

Our results revealed greater Glut-M activities during the mid-stance phase after long-term sand running (IG). Previous studies have shown that during the early phase of stance, the knee valgus is associated with hip adduction in individuals with OPF. This condition causes greater hip abductor activities, mainly due to the greater activity of the Glut-M muscle [37]. In addition, the weakness of the Glut-M muscles may increase the risk of sustaining injuries attributed to excessive subtalar pronation [11]. During running, the Glut-M muscle contracts to maintain lower limb alignment from the pelvis to the foot [38, 39]. However, the foot’s intrinsic muscle function is also critical in the mid-stance phase to avoid excessive pronation. The increased Glut-M muscle activity could be associated with reduced pronation, leading to new lower limb alignment and Glut-M muscle activation. Following our results and the previous study, we propose that sand running could be an effective rehabilitative means to treat lower limb injuries due to producing greater muscle activities [40].

The IG also showed significantly greater Gas-M activity at post-test than pre-test. To the authors’ knowledge, no study has examined the effects of long-term sand running on muscular activities in OPF individuals. However, a previous study showed that running on sand significantly increased the calf circumference over the training period. This condition may indicate a greater overload stimulus in that particular muscle group [41]. In addition, supporting our results, Pinnington et al. identified a significantly greater peak activation of the gastrocnemius when running on sand versus grass, primarily during the push-off phase of running, where there is plantar flexion of the foot [29]. Another previous investigation [42] reported that excessive rear-foot eversion during the stance phase of gait might result in increased internal rotation of the tibia with respect to the talus; associated joint coupling would cause the hip to internally rotate to a greater degree, thereby also increasing hip adduction and the dynamic Q angle [42]. Therefore, increasing the activity of Glut-M along with gastrocnemius may be increased the hip abduction and decrease the rear-foot eversion, respectively. Therefore, training on a continuously unstable bearing surface such as sand can improve the running mechanics by utilizing diverse muscle groups and increasing joint mobility. These results are consistent with a previous study indicating that athletic training on sand surfaces improved strength and endurance in the calf and thigh muscles [41].

Certain limitations of the present study must be acknowledged. First, the intervention group performed the exercise training on the sand, while the control group did not perform any exercise. This design cannot clarify which factor contributes to the changes in the EMG and foot posture index. That is, these changes might be due to just the (1) the intervened exercise irrespective of the ground surface (i.e., the observed training effect is due to the exercise type or not), (2) touching the sand irrespective of physical activities (i.e., surface-specific training effect or not), or (3) barefoot exercise itself irrespective of the ground surface or exercise types (i.e., barefoot-specific training effect or not). Second, we did not assess healthy control individuals and we could not say that the same protocols should be performed with the non-OPF runners. Therefore, further studies with different control groups are warranted to evaluate better the effects of exercise training on the sand on running biomechanics and muscle activities. Third, we examined the long-term effects of sand running only for active male individuals with OPF. Therefore, our results cannot be generalized to active female individuals. Future studies are needed to examine the long-term effects of sand running in females with OPF to establish whether sand is a preventive/rehabilitative means of reducing static foot pronation and improving muscle activities. Fourth, we did not examine running kinematics in the present study. Therefore, it is recommended for future studies to investigate the effect of sand running on kinematics and dynamic foot posture during running. Running kinematic assessment could reveal adaptations such as increased or decreased foot pronation and knee flexion to long-term sand running. Concerning the training studies on the sand to date, there is still a need for further research to determine the full range of physiological and biomechanical benefits associated with sand. The generalization should be considered carefully for the reasons described above.

## Conclusions

Long-term running on sand resulted in reduced pronation and increased Gas-M activity and improved frontal plane pelvic stability due to greater Glut-M activity in individuals with OPF. These findings can be attributed to the fact that training on sand requires more diverse muscles in individuals with OPF. Hence, we can confirm the potential of sand as a new training ground material when attempting to improve the walking ability, particularly the running mechanics in individuals with OPF. In addition, the increased hip and knee range of running on sand can be partly attributed to the increased EMG activation of the Gas-M and Glut-M muscles in individuals with OPF [7]. Although a complete evaluation and studies are necessary, our results can provide insights for researchers and clinicians to prevent or treat injuries in individuals with OPF, especially when dealing with RRIs in these individuals.

## Methods

### Participants

We used the G*Power and data in a previous study examining running muscle activities in individuals with OPF. The calculation parameters were power analysis of 0.05 (type I error) and 0.20 (type error II), i.e., 80% statistical power. Also considered were two tests (pre, post), the correlation coefficient of 0.5, and the effect size of 0.80 for running muscle activities (i.e., maximal tibialis anterior activity) [8]. As a result, 30 participants would be necessary to observe large group-by-time interactions.

In November 2019, individuals were recruited from physical therapy clinics in Ardebil city, Iran. An orthopedic surgeon assessed all individuals before selection. The eligibility criteria included a navicular drop of more than 10 mm [43], a foot posture index of > 10 [44], and a rearfoot striker. In the current study, a modified version of the navicular drop described by Brody [45] was used to determine the sagittal plane displacement of the navicular between the resting (seated) and stand on one leg positions. The participant was seated with both feet flat on the ground and knees flexed at 90°. The most medial aspect of the navicular was marked. A blank card was held at right angles to the foot against the navicular marking with the base of the card flat on the supporting surface. The height of the navicular was marked on the card. Then, the participant was asked to stand on one leg by flexing the contralateral knee. The single-limb stance position was selected, because recent work by McPoil and Cornwall [46] has shown that measurements taken from this position more accurately represent the position of the foot during the midstance phase of gait. A blank card was held at right angles to the foot against the navicular marking with the base of the card flat on the supporting surface. The height of the navicular was marked on the card. The difference between the height of the navicular in the resting (seated) and stand on one leg positions was recorded as the navicular drop*.* The foot posture index consists of six items to quantify and classify foot posture [44, 47]. These are (i) palpation of the head of the talus; (ii) curvatures above and below the lateral malleolus; (iii) position of the calcaneus in the frontal plane; (iv) prominence of the malleolus; (v) congruence of the medial longitudinal arch; and (vi) abduction/adduction of the forefoot. Each item was rated on a visual analog scale ranging from − 2 to + 2, resulting in a total score of − 12 to + 12. Negative values indicate supinated foot posture, and positive values indicate pronated foot posture. Of note, values of 10‒12 in the foot posture index were classified as over-pronated feet [44, 47]. The foot posture index was evaluated by a podiatrist with ∼10 years of professional experience. The validity of the foot posture index has been investigated fully and reported previously [44]. The foot posture index predicted 64% of the variance in static standing posture and 41% of the variance in the mid-stance posture during normal walking and demonstrated good inter item reliability (Cronbach’s α = 0.83) [44]. A more recent study has also demonstrated good internal construct validity and fit of the scoring system with Rasch model, a useful statistical model of the uni-dimensionality (capacity to measure a single construct) and scale stability (or linearity across a range of values) of a measure [48]. A detailed description of the foot posture index can be found elsewhere [44, 47]*.* In addition, the eligibility criteria were right-footed and physically active individuals with at least 1 year of experience of recreational running training with three sessions per week. The volunteers should also use the rearfoot strike to land the foot on the ground*.*

Individuals were removed from the study if they had limb length discrepancies of more than 5 mm, or reported muscle spasm, neuromuscular disorders, orthopedic-related diseases, or any previous surgery in the lower limbs and trunk. Only males were recruited for the present study, as previous studies showed distinct biomechanical characteristics between females and males [49, 50]. Females were associated with significantly greater knee abduction, knee internal rotation, and ankle eversion, while males were associated with substantially greater hip flexion [51]. Ferber et al. examined the gender differences in 3-D kinematics of the hip and knee. Female runners exhibited greater peak hip adduction, hip internal rotation, and knee abduction than men [52].

**Fig. 1:**
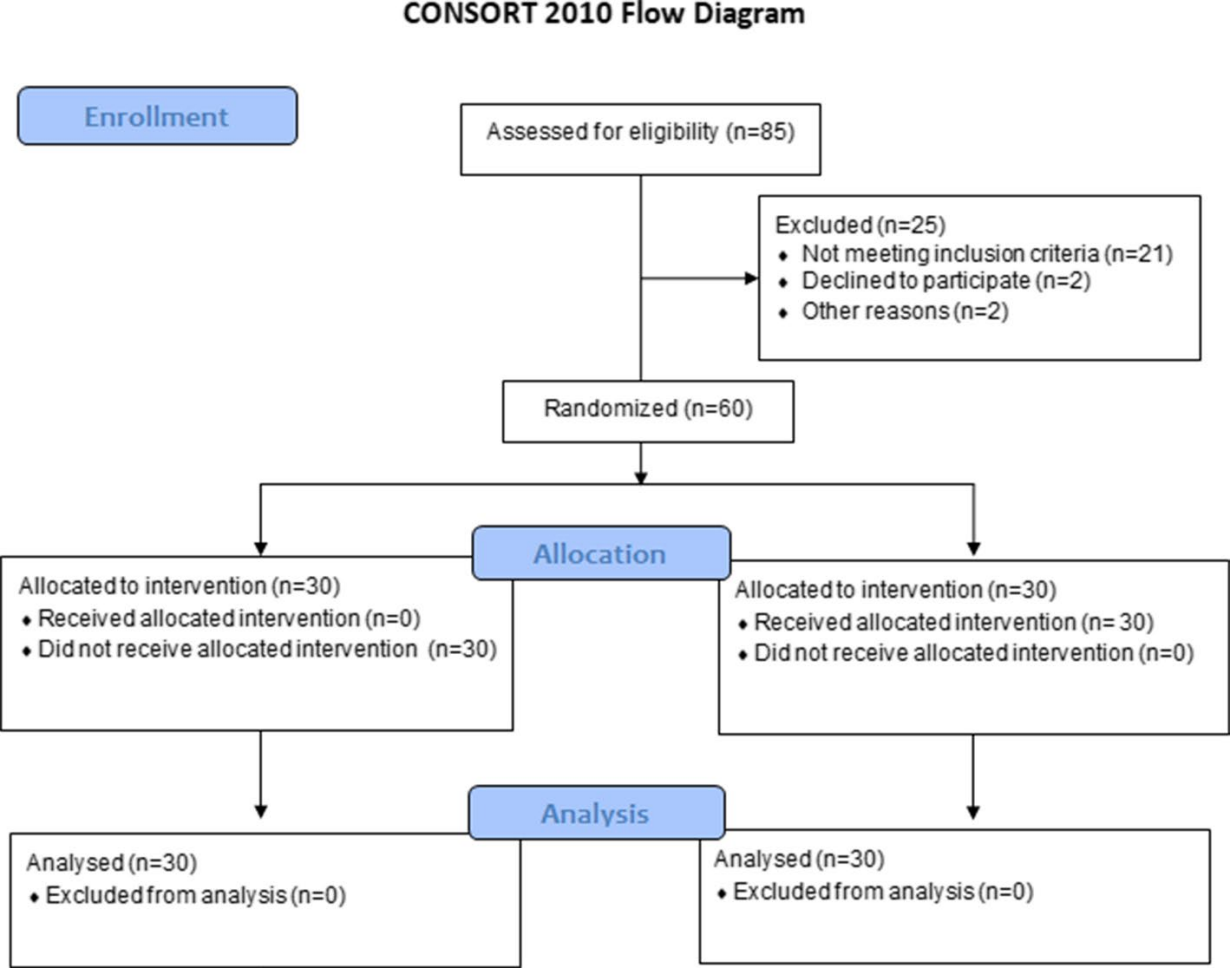
Flow diagram of the double-blinded randomized controlled trial

Eighty-five recreational, right-footed male runners with OPF were assessed; 25 were excluded (21 did not meet the eligibility criteria, two refused to participate in the study, and two for other reasons) (Fig. 1). Thus, 60 recreational male runners with OPF were randomly allocated to the intervention group (IG) (*n* = 30; age: 22.2 ± 2.5 years; height: 178.0 ± 6.6 cm; mass: 75.0 ± 8.2 kg) and control group (CG) (*n* = 30; age: 22.2 ± 1.9 years; height: 177.9 ± 5.7 cm; mass: 75.4 ± 7.9 kg).

The participants were randomized into the IG and CG. In addition, during the randomization process, a set of sealed, opaque envelopes was used to ensure the concealment of allocation. Thus, those evaluating the participants were unaware of the group allocation (i.e., blind assessors). All participants had their dominant foot ascertained using a kicking ball test and received an explanation of the procedures before obtaining informed consent.

### Experimental procedures

The participants performed a warm-up protocol including stretching the lower limb muscles and 5 min of self-selected running speed to familiarize themselves with the laboratory environment [53]. Next, all participants ran barefoot over an 18 m runway. Two sets of infrared photocells positioned 6 m apart along the length of the runway were used to monitor the running speed and set it at a speed of 3.3 m/s ± 5% [54]. The photocells were placed at approximately shoulder height to avoid being triggered by arm swing [55]. Running at this speed has previously been used for determining running-related risk factors of injuries [56]. A trial was considered successful if the dominant foot (the dominant limb was measured) landed in the force plate center and if the EMG signals were clean upon visual examination. Five successful running trials, both pre-test and post-test, were used for data analysis [29]. Maximum voluntary isometric contraction (MVIC) tests were then applied for each muscle to normalize the EMG data (Appendix 1).

### Experimental setup and data processing

A force plate (Bertec Corporation, Columbus, OH, USA) was located at the center of the level stable runway. A force plate with a sampling rate of 1000 Hz was used to register the GRF data. The description of the kinetic data process can be found in detail in a previous study [57]. The GRF data was processed using a low-pass filter at 20 Hz (4th order Butterworth filter, zero lag). The threshold of 10 N was used to determine the heel strike and toe-off.

**Fig. 2:**
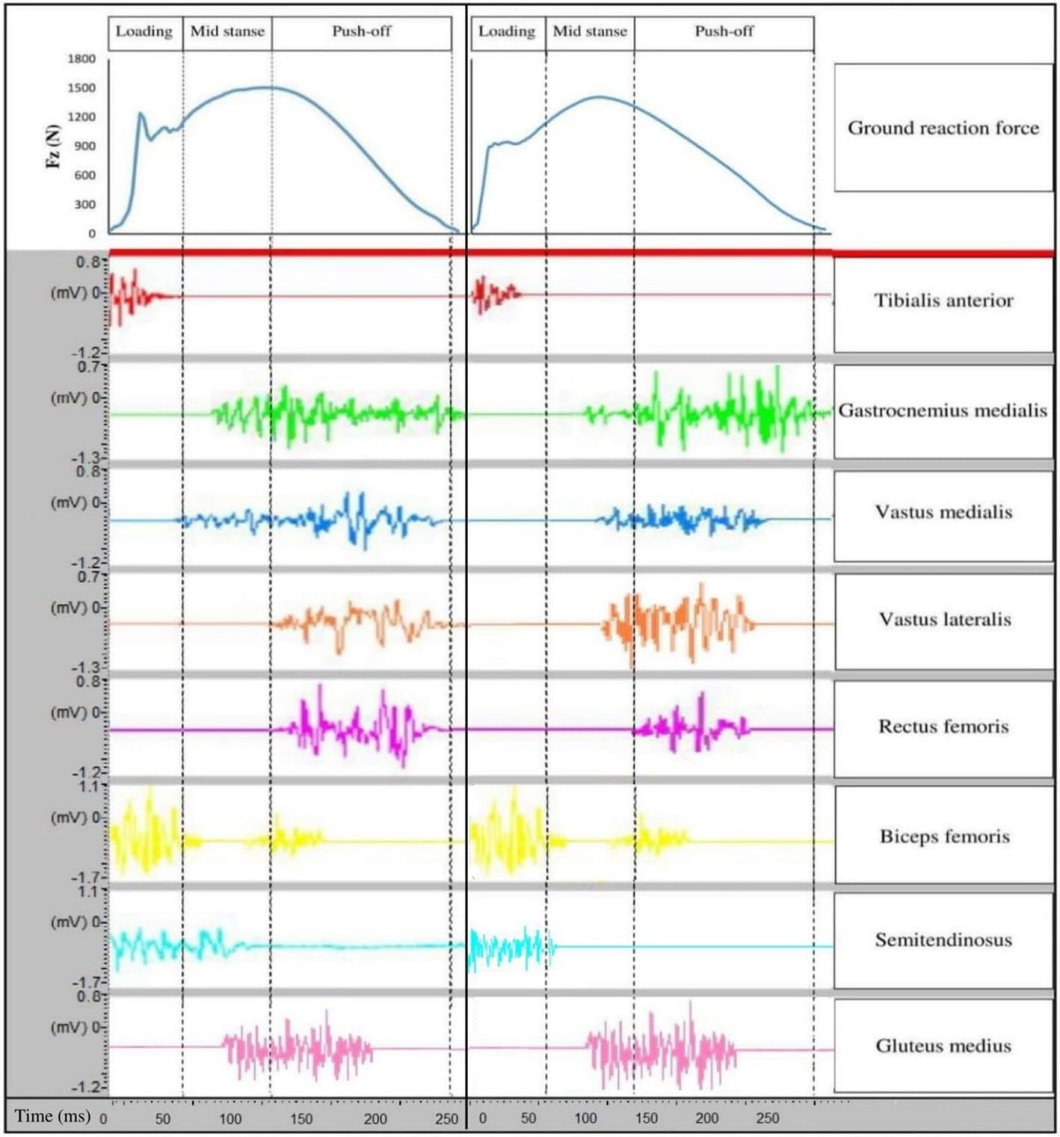
Loading, mid stance, and push-off phase of running during both pre-test (left side) and post-test (right side). Fz stand for vertical ground reaction force.

Eight pairs of bipolar Ag/AgCl surface electrodes (25 mm center-to-center distance; input impedance of 100 MΩ; and common-mode rejection ratio of > 110 dB) were used to register the muscle activities of tibialis anterior (TA), gastrocnemius medialis (Gas-M), biceps femoris (BF), semitendinosus (ST), vastus lateralis (VL), vastus medialis (VM), rectus femoris (RF), and gluteus medius (Glut-M) of the dominant leg [8]. The raw EMG signals were digitized at 1000 Hz using a wireless EMG system (Biometrics Ltd, Nine Mile Point Ind. Est., Newport, UK). Before electrode fixation, the skin was gently shaved, rubbed, and cleaned with alcohol. The Nexus software (Oxford Metrics, Oxford, UK) synchronized the GRF and EMG data, which was processed according to a previous study [37]. The run was divided into three phases to analyze the EMG data: the loading (0‒20% stance phase), mid-stance (20‒50% stance phase), and push-off (50‒100% stance phase) phases [58, 59, 60] (Fig. 2). All raw EMG data were processed in a custom program written in MATLAB (Release 12, MathWorks Inc.). First, raw EMG data were high-pass filtered using a zero-lag fourth-order recursive Butterworth filter (cutoff frequency 10 Hz) to remove movement artifacts. Then, full-wave was rectified and filtered using a low pass Butterworth filter (cutoff frequency of 6 Hz). The muscle activation profile was then normalized to the MVIC value for each respective subject. The aligned EMG data were then normalized to the stance (30 data points) and stride (51 data points), interpolated using a cubic spline, and then exported to Microsoft Excel. The following EMG parameters were then extracted for analysis: EMG of individual muscles expressed as a fraction of MVIC over the running phases [29].

### Sand running training protocol

**Fig. 3:**
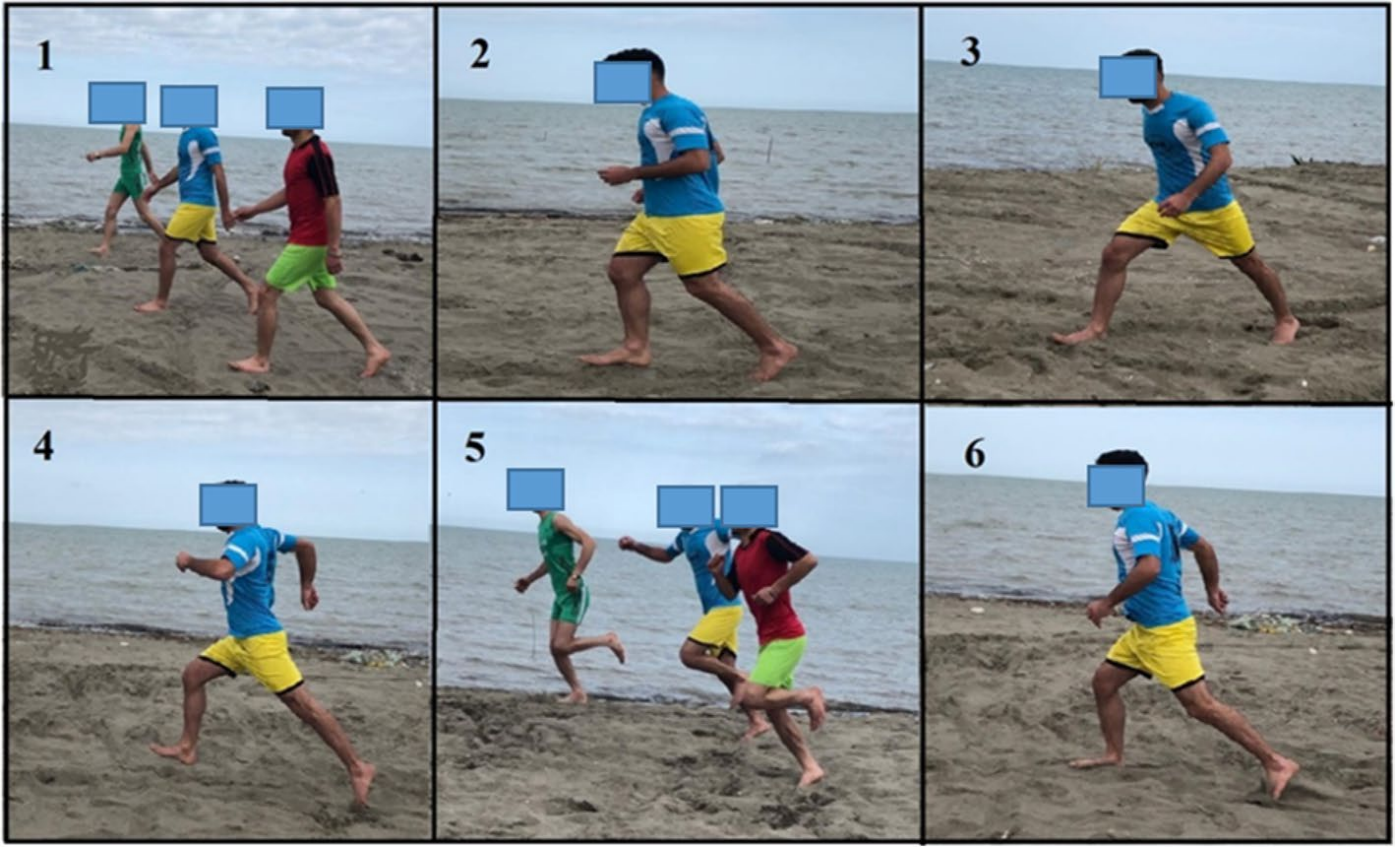
Examples taken from the progressively designed exercise program on sand. **1** walking exercise; **2** jogging exercise; **3** striding exercise; **4** bounding exercise; **5** galloping exercise; and **6** short sprints

The IG performed the sand running training program, including continuous jogging, striding, bounding, galloping, and short sprints, for 8 weeks (three sessions per week) [53, 61]. The training program was carried out in barefoot conditions. Each session was started with a warm-up and stretching session for 5 min and ended with a 5-min warm-down session [53]. Training duration was 50 min per session [53] (Table 5; Fig. 3). Participants started the stride by running at low speed, focusing on a short, quick stride for striding. After that, they gradually increased their speed using longer strides. During the exercise, individuals were asked to keep their torso extended and relaxed. For bounding, a straight-leg bound exercise was used to develop the power output of the lower limbs. For this purpose, participants started the exercise with their feet hip-width apart. After that, they started the first bound by sweeping the lead leg forward with the knee joint fully extended. At the same time, the opposite arm swept forward to match the range of the lead leg. During the following movement sequence, participants quickly pulled the lead leg back toward the ground with the foot in dorsiflexed position to prepare for a dynamic landing. During the exercise, participants were asked to realize midfoot ground contact with fully extended posture. The knee was extended during landing. Thereafter, participants moved the knee of the free leg forward to initiate the second straight-leg bound. They performed this cyclical bounding movement for a distance of 30 m. Galloping was performed with either foot as the lead. For this purpose, one foot was placed in front of the opposite foot. The front foot took a large step forward, while the second foot remained in place. After that, the back foot took a step forward but always remained behind the front foot. The 25 m sprints began with the participants in a forward lunge position. Time was started on the command "go" and stopped when the individual’s foot touched the finish line. The elapsed time was measured using a handheld watch with an accuracy of 0.1 s. Participants were instructed to perform the test at maximal effort and as fast as possible.

**Table 5:** Exercise protocol of the intervention group

Exercise	Duration (minutes)	Repetitions (number)	Distance (meters)	Recovery period (minutes)
Walking	5	–	50	–
Continuous jogging	20	–	50	–
Striding	3	2–3	50	1
Bounding	3	2–3	30	1
Galloping	3	2–3	30	1
Short sprints	6	3–5	25	2

A physiotherapist supervised each session to modify the exercise or the progression to meet the given training program and ensure the correct technique. We evaluated the IG after the intervention, scheduled 6 days after the final session. We used this procedure to avoid interference of acute physiological responses to training [62]. We also evaluated CG for the second time after 9 weeks, but the group participants did not receive any exercise. Individuals from the CG followed their regular daily routine and did not perform additional exercise during the intervention period. After the intervention period, individuals from the CG could receive the same exercise program as the IG. All participants were asked not to take up any extra physical activity or exercise during the experimental period. Table 5 illustrates the training exercises for the IG [53].

### Statistical analyses

The Shapiro‒Wilk test evaluated and confirmed the data normality, and the data were presented in mean and standard deviations. A mixed ANOVA (time: pre vs. post) × (group: CG vs. IG) was used to compare outcomes between groups over time. Post-hoc analyses were calculated using the Bonferroni test. We determined the effect sizes by converting partial eta-squared (η2p) to Cohen’s d (*d* < 0.50 indicate small effects, 0.50 ≤ *d* < 0.80 indicate medium effects, and *d* ≥ 0.80 indicate large effects). All analyses were performed using SPSS version 24.0, with a significance level set at *p* < 0.05.

## Data Availability

The data sets used and/or analyzed during the current study are available from the corresponding author on reasonable request.

## Appendix 1

Description of the maximum voluntary isometric contraction (MVIC) tests for tibialis anterior (TA), gastrocnemius medialis (Gas-M), biceps femoris (BF), semitendinosus (ST), vastus lateralis (VL), vastus medialis (VM), rectus femoris (RF), and gluteus medius (Glut-M) muscles.

**Table:**

Muscles	Test protocol
TA	In seated position on a chair with back rest, with 90° hip, knee, and ankle joint flexion. Participants were asked to activate TA at maximal effort against resistance
Gas-M	In seated position on the examination table with the hip flexed at 90° and the knee and ankle in neutral position. Participants activated their plantar flexors at maximal effort against resistance
BF	In seated position on a chair with hip and knee flexed at 90°. Participants activated the hamstring muscles at maximal effort against resistance
ST	In seating position on a chair with hip and knee flexed at 90°. Participants maximally activated their knee flexors against resistance
VL	In seated position on a chair with hip and knee flexed at 90°. Participants maximally activated their knee extensors against resistance

## Abbreviations

RRIs: Running-related injuries
OPF: Over-pronated foot
GRFs: Ground reaction forces
FM: Free moment
EMG: Electromyography
IG: Intervention group
CG: Control group
MVIC: Maximum voluntary isometric contraction
TA: Tibialis anterior
Gas-M: Gastrocnemius medialis
BF: Biceps femoris
ST: Semitendinosus
VL: Vastus lateralis
VM: Vastus medialis
RF: Rectus femoris
Glut-M: Gluteus medius
